# Systematic integrative analysis of gene expression identifies HNF4A as the central gene in pathogenesis of non-alcoholic steatohepatitis

**DOI:** 10.1371/journal.pone.0189223

**Published:** 2017-12-07

**Authors:** Cristina Baciu, Elisa Pasini, Marc Angeli, Katherine Schwenger, Jenifar Afrin, Atul Humar, Sandra Fischer, Keyur Patel, Johane Allard, Mamatha Bhat

**Affiliations:** 1 Multi Organ Transplant Program, University Health Network, Toronto, Canada; 2 Division of Gastroenterology and Hepatology and University of Toronto, Toronto, Canada; 3 Division of Pathology, University Health Network, Toronto, Canada; University of Navarra School of Medicine and Center for Applied Medical Research (CIMA), SPAIN

## Abstract

Non-alcoholic fatty liver disease (NAFLD) is the most common chronic liver disease in the Western world, and encompasses a spectrum from simple steatosis to steatohepatitis (NASH). There is currently no approved pharmacologic therapy against NASH, partly due to an incomplete understanding of its molecular basis. The goal of this study was to determine the key differentially expressed genes (DEGs), as well as those genes and pathways central to its pathogenesis. We performed an integrative computational analysis of publicly available gene expression data in NASH from GEO (GSE17470, GSE24807, GSE37031, GSE89632). The DEGs were identified using GEOquery, and only the genes present in at least three of the studies, to a total of 190 DEGs, were considered for further analyses. The pathways, networks, molecular interactions, functional analyses were generated through the use of Ingenuity Pathway Analysis (IPA). For selected networks, we computed the centrality using igraph package in R. Among the statistically significant predicted networks (p-val < 0.05), three were of most biological interest: the first is involved in antimicrobial response, inflammatory response and immunological disease, the second in cancer, organismal injury and development and the third in metabolic diseases. We discovered that HNF4A is the central gene in the network of NASH connected to metabolic diseases and that it regulates HNF1A, an additional transcription regulator also involved in lipid metabolism. Therefore, we show, for the first time to our knowledge, that HNF4A is central to the pathogenesis of NASH. This adds to previous literature demonstrating that HNF4A regulates the transcription of genes involved in the progression of NAFLD, and that HNF4A genetic variants play a potential role in NASH progression.

## Introduction

Non-alcoholic fatty liver disease (NAFLD) comprises a spectrum of liver disease, progressing from simple steatosis, to non-alcoholic steatohepatitis (NASH) with subsequent risks of developing NASH cirrhosis, and hepatocellular carcinoma[1]. NAFLD is now recognized as the most common cause of chronic liver disease, and is associated with an increasing prevalence of obesity and diabetes[2]. Studies have reported a prevalence of NAFLD ranging from 10 to 46% in the United States, and biopsy-based studies have documented the presence of NASH in 3–5%[3, 4]. NASH cirrhosis has become the second most common indication for liver transplantation[5], and is on track to become the most common indication for liver transplantation in the Western world, with the anticipated decrease in hepatitis C burden following successful antiviral therapy[6].

The diagnosis of NASH is suspected based on the presence of metabolic risk factors, steatosis on ultrasound, and elevated transaminases, in the absence of any other etiology of liver disease[7]. It is confirmed through liver biopsy demonstrating characteristic features of steatosis, ballooning, and lobular inflammation. NASH can regress with weight loss through exercise and diet, but few patients can adopt and maintain these lifestyle changes long-term. Although there are promising compounds in clinical trials, there are no approved therapies that alter long-term disease progression in NASH or improve advanced fibrosis [8]. Given the increasing burden of disease, there is a clear need for biomarkers to detect disease at an earlier stage, as well as identification of more optimal therapeutic agents.

The molecular mechanisms associated with NASH pathogenesis have become better understood over recent years. The two-hit hypothesis has been widely accepted as reflective of NASH pathogenesis. The two proposed hits include fat accumulation in the liver, followed by oxidative stress triggering ongoing inflammation. Pathways such as insulin resistance[9], lipid metabolism[10], apoptosis[11], endoplasmic reticulum stress[12], mitochondrial dysfunction[13] and immune response[14] have been found to contribute to NASH pathogenesis based principally on data from animal models. However, a holistic understanding incorporating the crosstalk of pathways and networks in driving NASH pathogenesis has been lacking. Therefore, the aim of our study is to perform an integrative analysis of all available high-throughput gene expression data on NASH in patients to elucidate the key genes and pathways involved in its molecular pathogenesis.

## Results

### Canonical pathways

**Fig 1:**
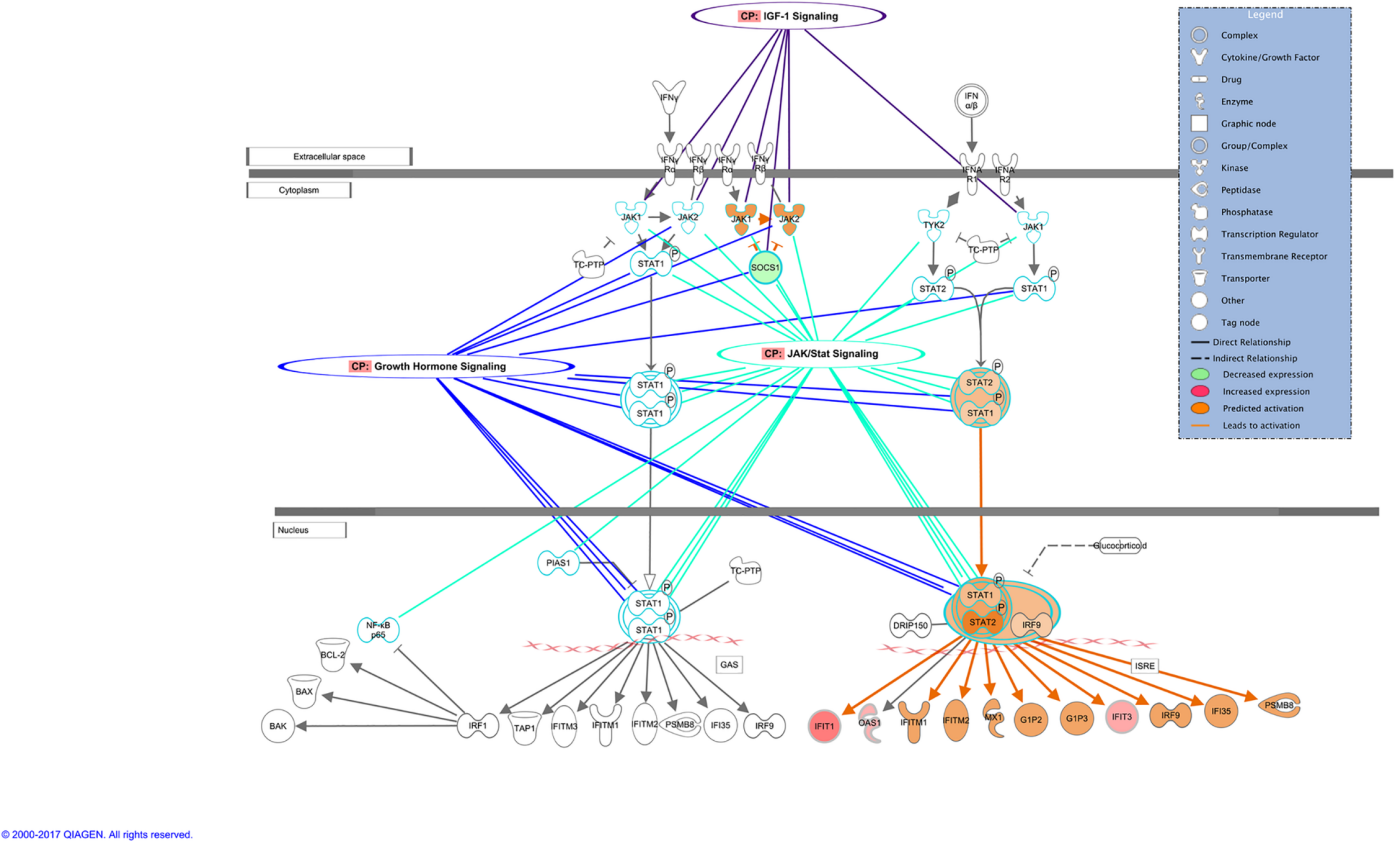
Interferon signaling pathway cross-talk with other activated pathways in NASH.

Based on the gene expression from our data, a set of predicted activated or inhibited pathways were identified. Table 1 lists the most significantly changed pathways due to the expression level of our input genes. Among the activated pathways in NASH, Interferon signaling, JAK/Stat Signaling and IGF-1 Signaling appear to be most significantly affected, which is in agreement with previous reports in the literature[15, 16]. In the Interferon Signaling pathway, shown to crosstalk with other activated pathways (Fig 1), the decreased expression of SOCS1, a suppressor of cytokine in cytoplasm, leads to activation of JAK1/2 kinases. On the other hand, upregulation of IFIT1, IFIT3 (interferon-induced proteins 1, 3) in nucleus predicts the activation of other molecules, e.g.; IFITM1, IFITM2 (interferon-induced transmembrane proteins 1, 2), IRF9 (interferon regulatory factor 9), and IFI35 (interferon-induced protein 35) in the Interferon Signaling pathway. Not surprisingly, several genes are shared among the activated pathways, e.g.; SOCS1, SOCS2, FOS, FGR2. The second half of Table 1 presents the inactivated canonical pathways. p53 Signaling, ERK/MAPK Signaling and Telomere Signalling, shown to be activated in cancers[17, 18], here are predicted to be inhibited, since most of the genes within those pathways are downregulated. As for the activated pathways, they also cross-talk through common molecules (S1 Fig). In addition, mTOR Signaling is also predicted to be inactivated in NASH, although the literature does support its activation in lipogenesis resulting in steatosis[19]. We hypothesize that for our data, the downregulation of the proto-oncogenes and thus, of the corresponding cancer pathways, could be explained by the upstream regulator analysis presented below.

**Table 1:** Significant pathways predicted to be activated or inhibited by z-score. A positive z-score predicts activation, a negative z-score predicts inhibition. Genes listed in Bold and *Italic* show up- and down-regulation, respectively.

Canonical Pathways	-log(p-value)	zScore	Input genes
Interferon Signaling	3.55E+00	2.000	**IFIT1**, *SOCS1*, **IFIT3, OAS1**
JAK/Stat Signaling	3.07E+00	1.342	*FOS*, *SOCS1*, *CDKN1A*, *SOCS2*, **FGFR2**
IGF-1 Signaling	4.35E+00	1.000	*FOS*, *SOCS1*, *SOCS2*, **FGFR2,** *IGFBP1*, *CYR61*, *IGFBP2*
Growth Hormone Signaling	2.23E+00	1.000	*FOS*, *SOCS1*, *SOCS2*, **FGFR2**
STAT3 Pathway	4.34E+00	0.816	*MYC*, *SOCS1*, *CDKN1A*, *RAC1*, *SOCS2*, **FGFR2**
p53 Signaling	2.51E+00	-2.000	*GADD45B*, *THBS1*, *CDKN1A*, **FGFR2,** *GNL3*
ERK/MAPK Signaling	2.06E+00	-1.633	*MYC*, *FOS*, *ELF3*, *ETS2*, *RAC1*, **FGFR2**
Telomerase Signaling	2.51E+00	-1.000	*MYC*, *ELF3*, *ETS2*, *CDKN1A*, **FGFR2**
mTOR Signaling	1.49E+00	-0.447	*NAPEPLD*, *RHOB*, *RAC1*, **FGFR2, PLD1**

### Upstream regulator analysis

**Fig 2:**
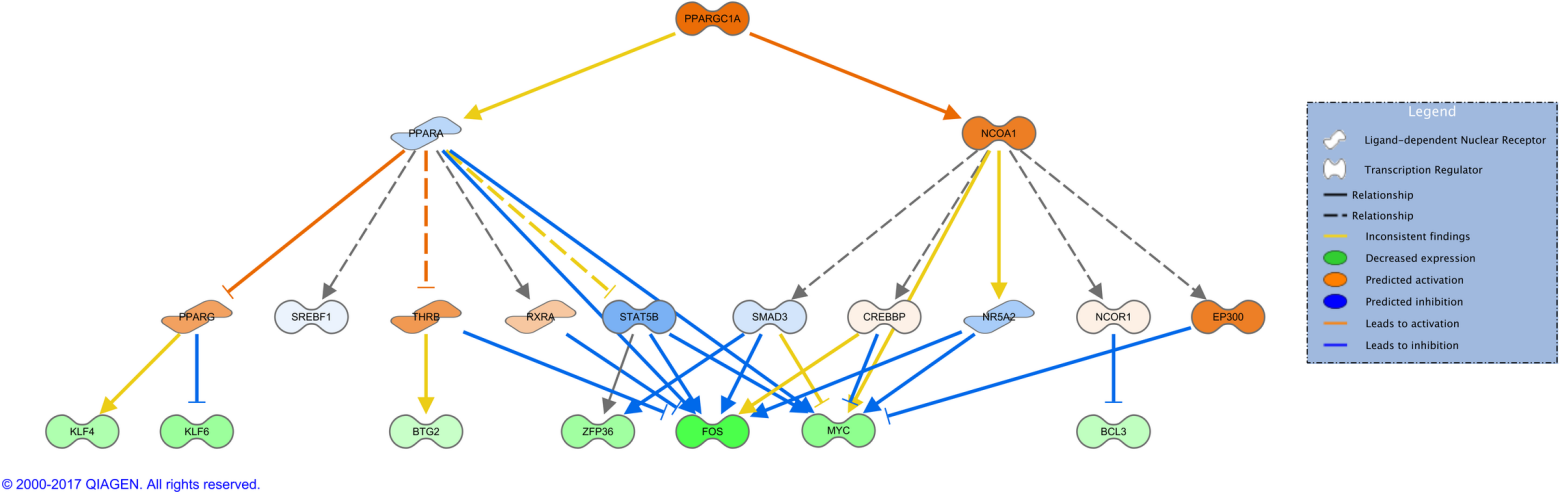
Mechanistic network of PPARGC1A.

IPA uses experimentally-observed relationships between regulators and genes in the dataset to predict upstream transcriptional regulators. The calculated z-score predicts either activation or inhibition of regulators, based on the relationship with dataset genes and direction of change of input genes. There are twelve activated regulators of various categories, with three transcription regulators (IRF3, ZBTB20, PPARGC1A), a cytokine (IFNB1), a transporter (SFTPA1), two drugs (stallimycin, bromodeoxyuridine), as listed in Table 2. For instance, the protein encoded by PPARGC1A (PPARG coactivator 1 alpha) is known to bind and regulate many genes involved in energy metabolism[20]. It interacts with PPARγ and PPARα, which in turn mediate interactions with multiple transcription factors[21]. A mechanistic network that could explain how the upstream molecules such as PPARGC1A drive the observed expression changes is shown in Fig 2. The network is hierarchically organized on multiple levels: on top lays the upstream regulator, in this case PPARC1A that regulates PPARα and NCOA on the second level (both part of PPAR signaling pathway with established roles in NAFLD[22, 23]); the third layer is represented by a series of ligand receptors or transcription factors (TFs) regulated by PPARα and NCOA, and the last row represents the selection of genes from our data set. From a total of 13 input genes that could interact with this mechanistic network, we selected seven that also represent transcription regulators. It is very noticeable that they are all downregulated, most likely due to inhibition from TFs in the layer above. The two oncogenes FOS and MYC that can also be directly inactivated by PPARα. The blue lines indicate direct inhibition, leading to a lower gene expression of the corresponding genes.

**Table 2:** Upstream regulators predicted to be activated.

Upstream Regulator	Molecule Type	z-score	p-value of overlap
Hdac	group	2.923	1.03E-05
SFTPA1	transporter	2.881	4.78E-06
DUSP1	phosphatase	2.276	2.16E-03
PLAU	peptidase	2.168	1.98E-06
2-amino-5-phosphonovaleric acid	chemical—other	2.11	1.87E-03
Immunoglobulin	complex	2.101	3.39E-04
IRF3	transcription regulator	2.062	1.97E-03
ZBTB20	transcription regulator	1.969	1.16E-03
stallimycin	biologic drug	1.842	3.51E-06
bromodeoxyuridine	chemical drug	1.838	8.16E-06
IFNB1	cytokine	1.758	1.10E-06
PPARGC1A	transcription regulator	1.641	1.08E-03

**Fig 3:**
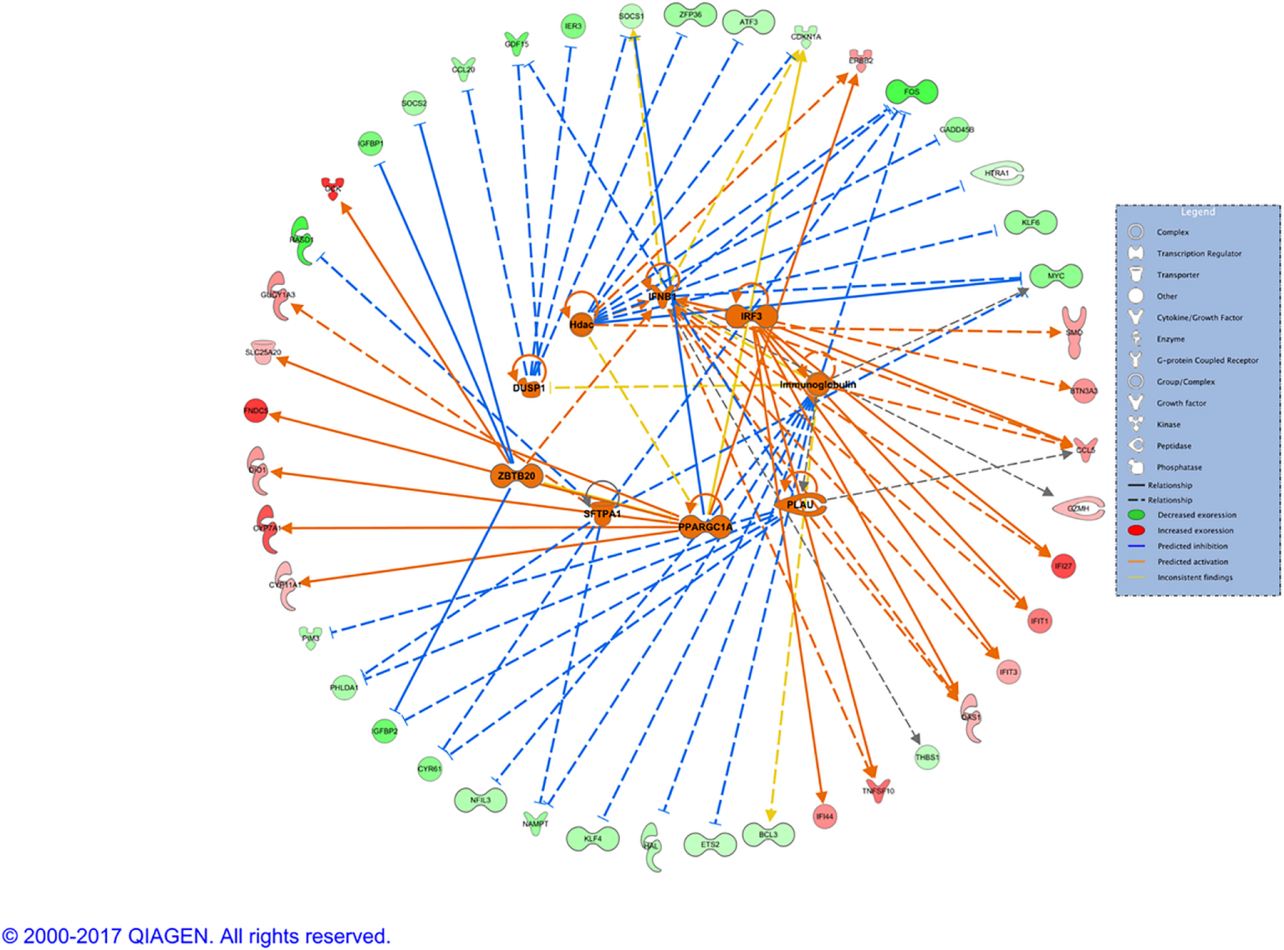
Network of predicted activated upstream regulators.

The activated upstream regulators (except for the drugs) and their interactions with the input genes are presented in the form of a network in Fig 3. They fall into two categories: one leading mostly to the activation of the genes (IFNB1, IRF3, PPARGC1A) with higher level of expression, and one leading mostly to the inactivation of the molecules (HDAC, DUSP1, ZBTB20, SFTPA1, Immunoglobulin) resulting in a low expression level. The list of inhibited upstream regulators is much larger and consists of cytokines, growth factors, complexes, transcription regulators, chemicals, etc (S1 Table). Here, we focus on the transcription regulators that are inactivated and their interactions, as well as with genes in our dataset (S2 Fig). As per graphical representation, the inhibition of the upstream regulators influences the level of the expression of the downstream genes, the majority of which are downregulated, e.g.TP63, EGR1, CREB1, etc.

### Diseases and functions

**Fig 4:**
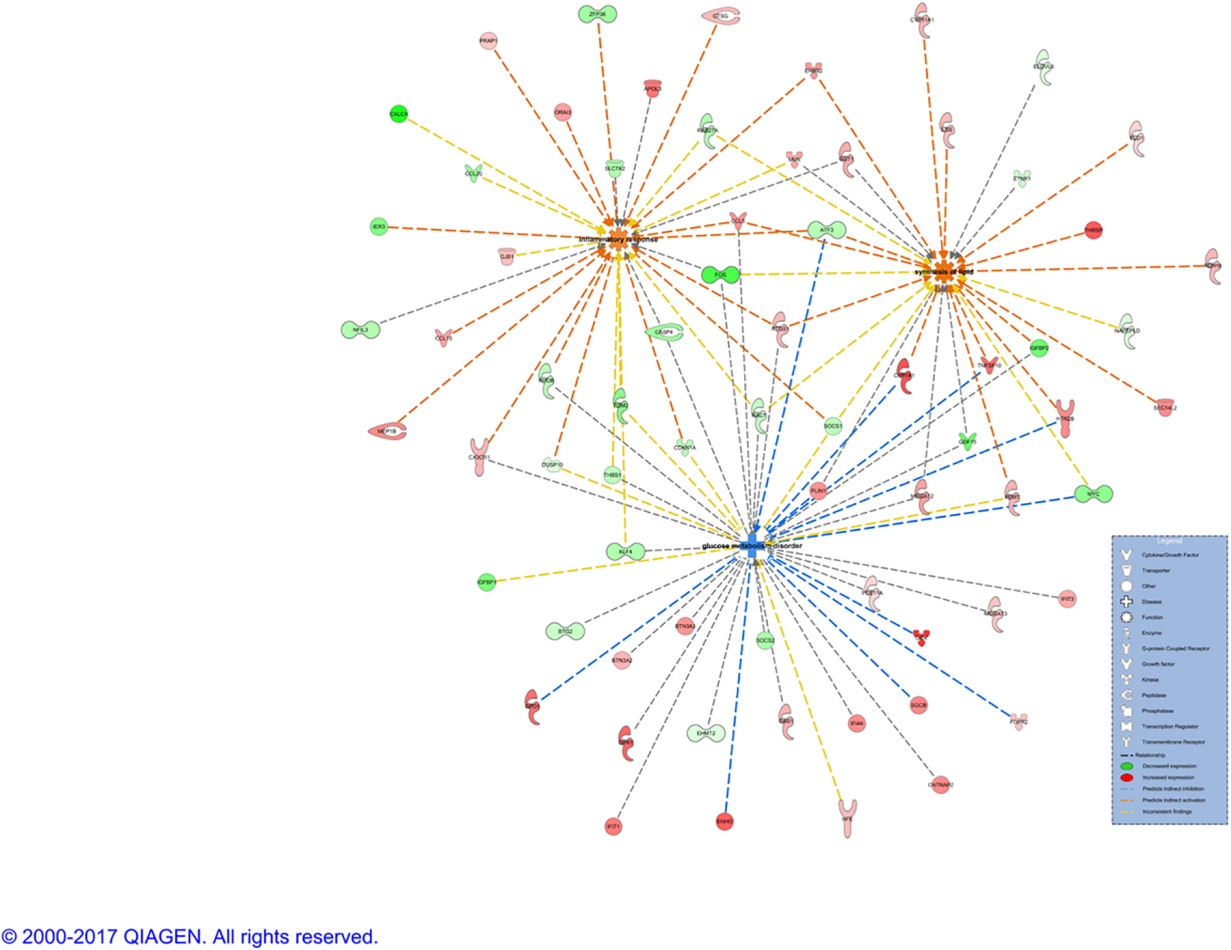
Network of inflammatory response, glucose metabolism and lipid metabolism in NASH.

Not surprisingly, for gene expression data from patients with NASH, the top diseases and biological functions are: cancer, inflammatory response, organismal injury and abnormalities, metabolic diseases. The corresponding p-value and the number of molecules are presented in Table 3. The inflammatory response and lipid synthesis are predicted to be enhanced, whereas the glucose metabolism disorder is reduced in NASH (Fig 4). The subcellular view of lipid metabolism and its functions relative to the gene expression level in our dataset is presented in greater details in S3 Fig.

**Table 3:** Top diseases and functions.

Name	p-value range	# Molecules
Cancer	1.07E-03–5.06E-09	178
Organismal Injury and Abnormalities	1.04E-03–5.06E-09	182
Tumor morphology	1.04E-03–5.74E-09	32
Inflammatory response	8.14E-04–6.11E-09	77
Metabolic disease	2.65E-04–7.56E-09	45

### Networks

**Fig 5:**
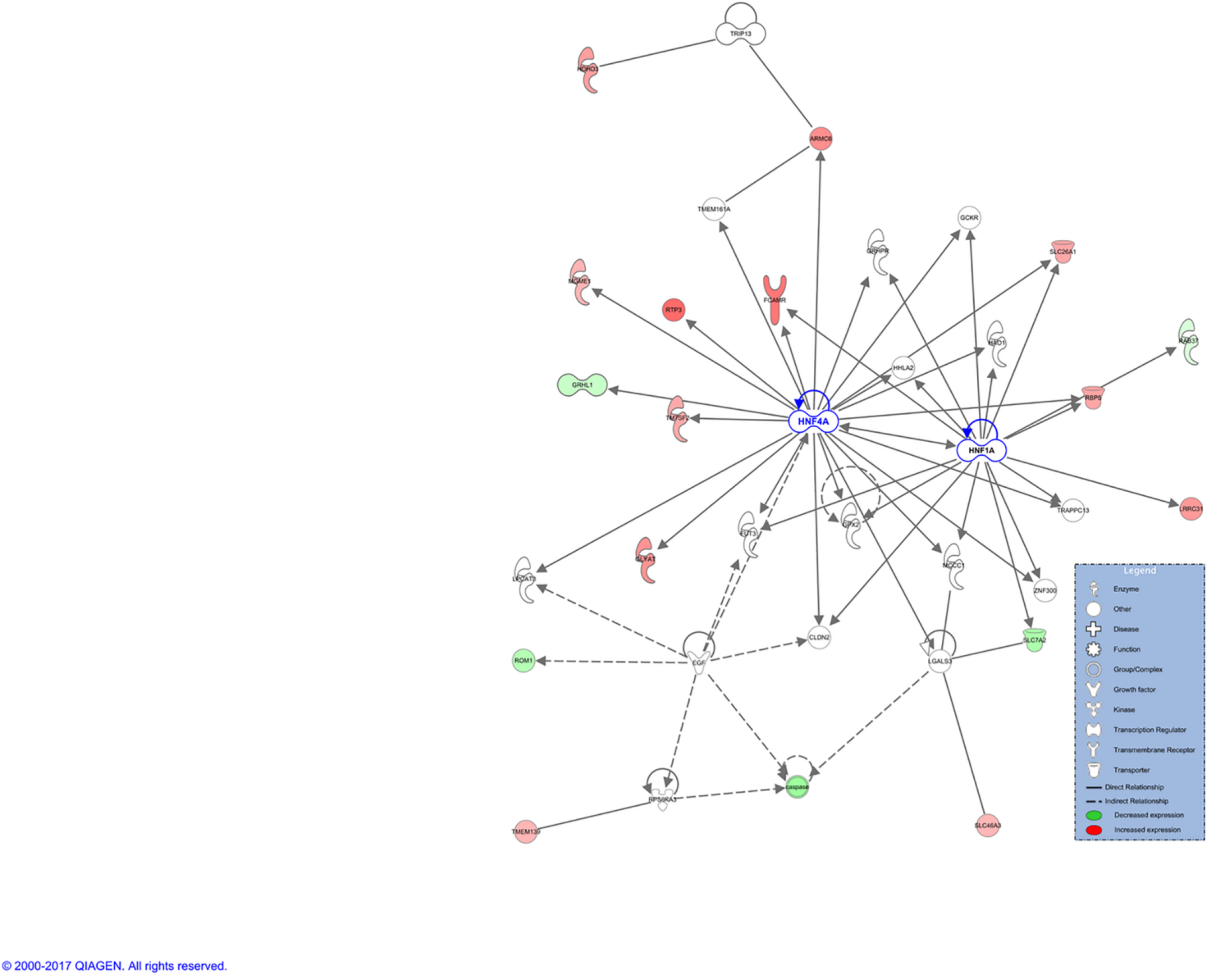
Network associated with glomerular injury, metabolic disease, organismal injuries and abnormalities: Illustration of HNF4A as central gene.

Based on input data set, 15 networks were identified and listed in decreasing order of significance (S2 Table). Three of them are of relevant biological importance for our data and are associated with the following diseases and functions: i) antimicrobial response, inflammatory response, immunological disease; ii) cancer, organismal injury and abnormalities, cellular development, and iii) glomerular injury, metabolic disease, organismal injury and abnormalities. For the first network (S4 Fig), the central nodes, in order of decreasing of betweenness score are: NFkB (complex) (score = 295.5), Mir122a, b (score = 93,7) and IFN Beta (score = 89.5). This is in agreement with previous findings, since NFkB (complex) is part of the NFkB (family) with known roles in expression, apoptosis, proliferation, cell death, differentiation, etc. in various cancers, including liver cancer[24], abdominal, epithelial cancer, inflammation, diabetes[25], etc. The microRNAs miR-122, together with miR-133-a, miR-134-a and miR-24, conveniently detectable also in serum and plasma, have recently been reported as biomarkers for hepatic inflammation, contributing to NASH development[26, 27]. Similar roles, plus activation in various infections by viruses are reported for IFN Beta[28]. However, none of these central nodes were present in the input gene expression data, but rather added by the Ingenuity Knowledge Base as interactors. The second network (S5 Fig), involved in cancer, has MYC as the central node (score = 144.3), followed by CDKN1A (score = 119.5) and FOS (score = 118.4). The two oncogenes, MYC and FOS, and the tumor suppressor, CDKN1A, are all downregulated in our NASH data and their level of expression was already discussed in the section above. The last network analyzed here and most biologically meaningful for NASH, is associated with metabolic diseases (Fig 5). The main player, aka central node, HNF4A (score = 409.5) is in physical interaction and regulating HNF1A, the second central molecule (score = 127.5). It has been established that the nuclear transcription factor nuclear factor 4 alpha (HNF4A) regulates the expression of HNF1A[29], another transcription regulator also involved in lipid metabolism. Additionally, genomic mutations in HNF4A could lead to diabetes[30], a condition frequently associated with NASH. Our results are in agreement with a previously described network of transcription factors regulated by HNF4A, involved in regulating hepatic fatty acid metabolism[31]. As can be graphically noticed, the majority of the molecules from this network are upregulated, suggesting activation of the entire network. These include a series of enzymes (GLYAT, TM7SF2, MGME1, HDHD3) and transporters (SLC26A1, SLC46A3, RBP5, RTP3) in the cytoplasm. When overlaying this network with canonical pathways in NASH, HNF4A and HNF1A are connected to hepatic cholestasis, diabetes and FXR/RXR activation (S6 Fig). HNF4A was found to be significantly deregulated (upregulated, log_2_(FC) = 0.772) in only one of the datasets used in this study (GSE89632), although this dataset represented the largest number of patients. HNF4A was therefore not included in our input list of DEGs for the integrative analysis, as per S4 Table. Nonetheless, HNF4A was the central protein in the significant network 3 derived by IPA, based on previous experimental and theoretical knowledge on protein-protein interactions. In order to assess the level of HNF4A expression, we performed experimental validation of HNF4A expression on NASH samples. This was done by immunohistochemistry (IHC) in 12 NASH patients with different NAFLD Activity Score-NAS (score 5–7). Digital quantification of tissue slides from healthy liver (control) and NASH samples reported the following percentages of HNF4A+ cells: 17% in healthy liver and a range of positivity between 24 and 40% in NASH patients. In particular, the percentage of strong positive reaches the highest score (42%) in patients with NAS score 7 compared to 22–33% strong positive for patients with NAS between 5 and 6. to drop to 8% in the healthy liver. The S7 Fig provides 2 patients representative of this trend of higher gene expression with increasing NAFLD Activity Score. This concords with our findings at the gene expression level, where higher level of HNF4A are identified in NASH samples compared to normal tissue.

## Discussion

NASH is clearly a multifaceted disorder, with multiple genes and pathways contributing to its development. A better understanding of the complexity of genes and pathways in NASH will help in identification of better biomarkers and therapeutic agents. In this study, we performed an integrative analysis of all publicly available high-throughput gene expression data in human NASH. This allowed us to obtain insights into the key drivers of NASH pathogenesis, specifically HNF4A as the central gene.

We found inflammatory pathways such as interferon signaling, JAK/Stat Signaling, and IGF-1 signaling to be the most critical to the development of NASH. They are predicted to be activated and cross-talking at different subcellular levels, with many input genes being upregulated. These results, although not new, re-affirm the key role of the innate immune activation in NASH[32]. A large number of innate immune cells are found in liver, including Kupffer cells, natural killer T cells and natural killer cells that enable the liver to respond to external pathogens, thus a high level of inflammation is encountered when these cells are overly activated[33]. Hepatic gene expression in NASH have also identified tissue remodeling/repair genes regulating matrix molecules, as expected for fibrotic injury[34]

HNF4A was identified as the gene central to NASH pathogenesis, and Its importance was further confirmed through validation in patient samples. Our validation revealed significantly upregulated HNF4A expression by immunohistochemistry in NASH samples as compared with healthy liver. An additional interesting finding was the increasing positivity and intensity of nuclear staining of this transcription factor with increasing NAFLD score in the NASH samples. These findings suggest HNF4A as a potential therapeutic target in NASH.

HNF4A is known to modulate regulatory elements in the promoters and enhancers of genes involved in cholesterol, fatty acid and glucose metabolism[35]. Specifically in the liver, HNF4A activates hepatic gluconeogenesis[36] and regulates the expression of several genes, including apolipoproteins[37]. Additionally, it regulates gene expression in pancreatic β cells to achieve glucose homeostasis[35, 38] and activates insulin genes both directly and indirectly[38, 39]. A recent study by Lake et al.[40] assessed the role of major transcription factor binding sites in NASH. HNF4A mRNA expression was significantly decreased in human NASH samples, suggesting the contribution of HNF4A to NAFLD development. Using knockout models, previous mouse studies have revealed the critical role of HNF4A in the control of bile acid synthesis and lipid homeostasis [41, 42]. The disruption of HNF4A in mouse liver induces failure of lipid metabolism[42]. In addition, an integrative analysis of NAFLD signatures in human and genetically modified mouse models[43] demonstrated that HNF4A as a transcription factor plays an important role in regulating the expression of the genes involved in the progression of NAFLD to hepatocellular carcinoma. Loss-of-function mutations in HNF4A gene have been shown to cause a monogenic form of type 2 diabetes as well as type 1 maturity-onset diabetes of the young (MODY1)[44]. Additionally, HNF4A has been associated with late-onset T2D in several populations[45, 46]. In our study, HNF4A is present in the network of NASH connected to metabolic diseases and regulates HNF1A, an additional transcription regulator also involved in lipid and amino acid metabolism. Moreover, when overlaying with other canonical pathways in NASH, HNF4A and HNF1A are linked with to hepatic cholestasis, diabetes and FXR/RXR activation. FXR agonists are already in late stage clinical development for NASH[47]. Recently, it has been suggested specifically that HNF4A could potentially be targeted for preventive therapies[40].

The key limitation of our study is the small number of NASH datasets in humans publicly available for integrative analysis, and the paucity of associated clinical data. Nonetheless, our study represents a systematic integration of all publicly available high-throughput gene expression data in NASH, which is valuable in providing direction with respect to the diagnostic and therapeutics in NASH.

In summary, we show for the first time to our knowledge, that HNF4A is central to the pathogenesis of NASH. This adds to previous literature demonstrating that HNF4A regulates the transcription of NAFLD progression genes, and that HNF4A genetic variants play a potential role in NASH progression. Further validation studies to assess the centrality of HNF4A to NASH pathogenesis are needed.

## Materials and methods

### Gene expression analysis

We performed an integrative computational analysis of publicly available gene expression data in NASH from GEO[48] (GSE17470, GSE24807, GSE37031, GSE89632). Identification of the differentially expressed genes (DEGs) for each data set was done using GEOquery[49]. The comparison was consistently performed between NASH patients and healthy controls (S3 Table), excluding samples with simple steatosis where available. A gene was considered differentially expressed if the FDR[50] adjusted p-value was less than 0.05, fold change (FC) greater than 1.5 or less than 0.5. We have identified only 22 DEGs common to all four data sets, which is too small to provide enough information for an integrative study such as this one. On the contrary, if we consider all the DEGs identified in all four studies regardless of the commonality, we end up with 4378 DEGs, a prohibitively large number. Therefore, we decided to consider the genes present in at least three out of four studies to a total of 190 DEGs as a cut-off for further integrative analysis, and calculated mean value of the log_2_(FC) across the data sets (S4 Table). Remarkably, all the DEGs had consistent deregulation sense (up or down-regulation) in all of the GSE studies they were present in. *Note*: Of all studies including gene expression in human NASH vs controls, we excluded from our analysis the array express dataset, E-MEXP-3291[51] due to non-standard classification of NASH samples. In addition, we could not identify any significantly DEGs in the other two datasets (GSE48452, GSE63067) to consider for further investigation.

### Experimental validation of HNF4A expression

This protocol was approved by the University Health Network Institutional Review Board. Twelve NASH patients were included for HNF4A IHC validation using anti HNF4-Alpha, mouse monoclonal antibody (Abcam, ab201460, rabbit monoclonal, clone EPR16885-99). FFPE sections were (5um) pre-treated for antigen retrieval following manufacturer’s instruction. The dilution for HNF4-alpha antibody was 1:2000 and an anti-rabbit was used as the secondary antibody. The complex was then visualized with hydrogen peroxide substrate and 3, 3’- diaminobenzidine tetrahydrochloride (DAB) chromogen. The slides were then counterstained with Harris Hematoxylin. The degree of HNF4-Alpha staining in each NASH case and healthy liver (control) was assessed using Imagescope software. Entire slides were digitally scanned by an Aperio ScanScope CS scanning system and analyzed by Aperio ImageScope Viewer software (Aperio Technologies Inc., Vista, CA) using the Positive Pixel Count v9 algorithm.

### Pathway and network analysis

The integrative analyses were generated through the use of IPA (Ingenuity Systems^®^, www.ingenuity.com). We uploaded the file with 190 gene IDs and the associated mean(log_2_(FC)) for a core analysis that consists of several modules: identification of canonical pathways, upstream regulation, association with diseases and functions, regulator effects and network analysis. IPA uses its own, manually-curated knowledge database, derived from experiments and findings published in top peer-reviewed journals. For each pathway and network, the p-value and a z-score were calculated. The p-value was determined by a right-tailed Fisher’s exact test[52] that takes into account the number of so-called focus molecules (input genes) in the network and the total number of molecules in the Ingenuity Knowledge Base that could be included in networks. The prediction of activation or inhibition is based on the z-score. The algorithm is designed to reduce the chance that random data will generate significant predictions. The graphical representation of a pathway/network consists of the nodes (genes) and edges (biological relationship between nodes), with characteristic symbols for the different types of molecules. The central nodes were identified using igraph package[53] (version 1.01) in R[54] (version 3.3.2), which calculates the betweenness score by computing the shortest paths between all the pairs of nodes in the network.

All clinical information for the NASH and Control patients in our integrative analysis are presented in the S5 Table.

## Supporting information

S1 Fig Telomere signaling cross-talk with other inactivated pathways in NASH. (TIF)

S2 Fig Network of predicted inhibited upstream regulators. (TIF)

S3 Fig Subcellular view of the lipid metabolism network. (TIF)

S4 Fig Network 1 associated with antimicrobial response, inflammatory response and immunological disease. Molecules in bold represent the central nodes.
(TIF)

S5 Fig Network 2 associated with cancer, organismal injuries and abnormalities, cellular development. Molecules in bold represent the central nodes.
(TIF)

S6 Fig Overlay of network 3 with canonical pathways in NASH. Molecules in bold represent the central nodes. CP = canonical pathway.
(TIF)

S7 Fig HNF4A expression in NASH tissues from NASH patients presenting different NAS score. A, B immunohistochemical analysis of liver sections from NASH patients (A, pts#1 NAS score = 7, B pts#12 NAS score = 5), magnification 40X. Sections were stained with HNF4A-DAB and counterstained with hematoxylin. C, D digital markup of sections depicted in column A and B, (C, pts#1 NAS score = 7, D pts#12 NAS score = 5) elaborated by Positive Pixel Count v9 algorithm using Aperio ImageScope image analysis software; blue = negative; yellow = weak positive; orange = positive; brown = strong positive. E, F pie charts represent the digital quantification of percentage of strong, medium and weak positive pixel quantified over the total of positive HNF4A pixel in the whole section (E, pts#1 NAS score = 7; F, pts#12 NAS score = 5).
(TIF)

S1 Table Upstream regulators predicted to be inactivated. (DOCX)

S2 Table IPA predicted networks associated with NASH. (DOCX)

S3 Table Distribution of healthy control and NASH samples from GEO data sets. (DOCX)

S4 Table List of DEGs and the mean log_2_(FC). FC = fold change. (DOCX)

S5 Table Clinical information of NASH and control patients from GEO studies. (DOCX)
