# Supplementary figures and images for: Systematic integrative analysis of gene expression identifies HNF4A as the central gene in pathogenesis of non-alcoholic steatohepatitis
Source: PLoS One. 2017 Dec 7;12(12):e0189223. doi: 10.1371/journal.pone.0189223 (PMC5720788; doi:10.1371/journal.pone.0189223)

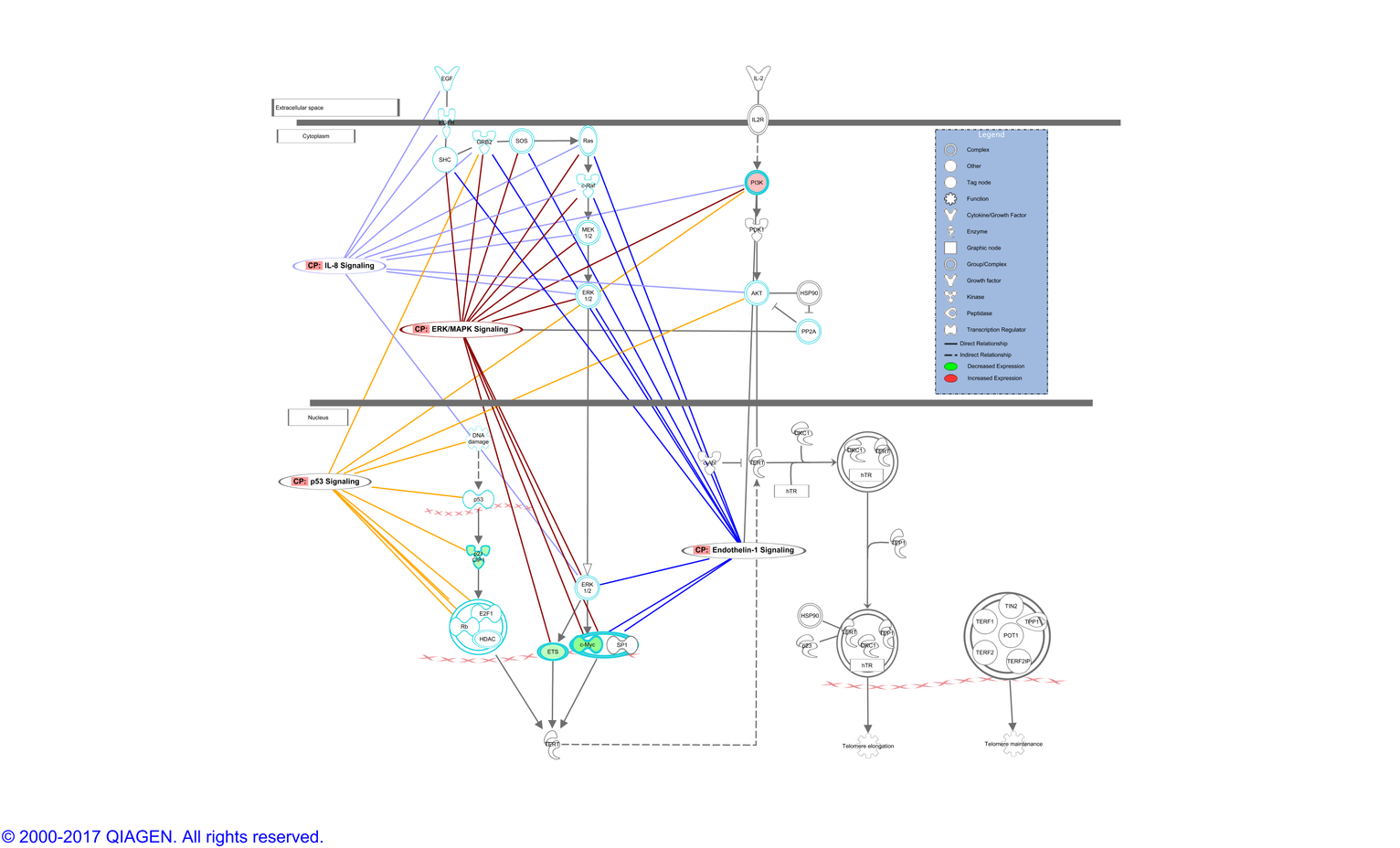

Supplement: S1 Fig — (TIF) [file pone.0189223.s001.tif]

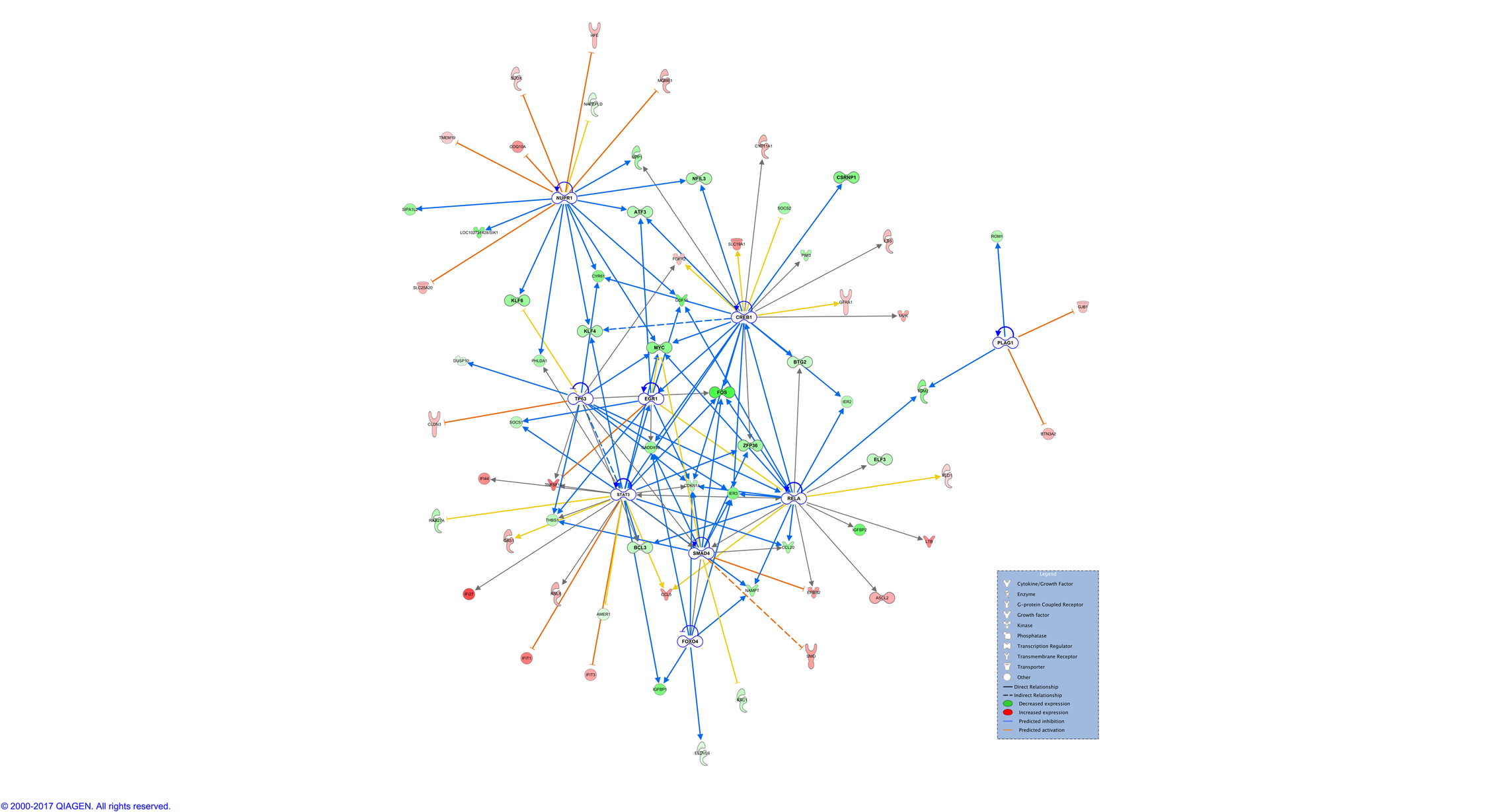

Supplement: S2 Fig — (TIF) [file pone.0189223.s002.tif]

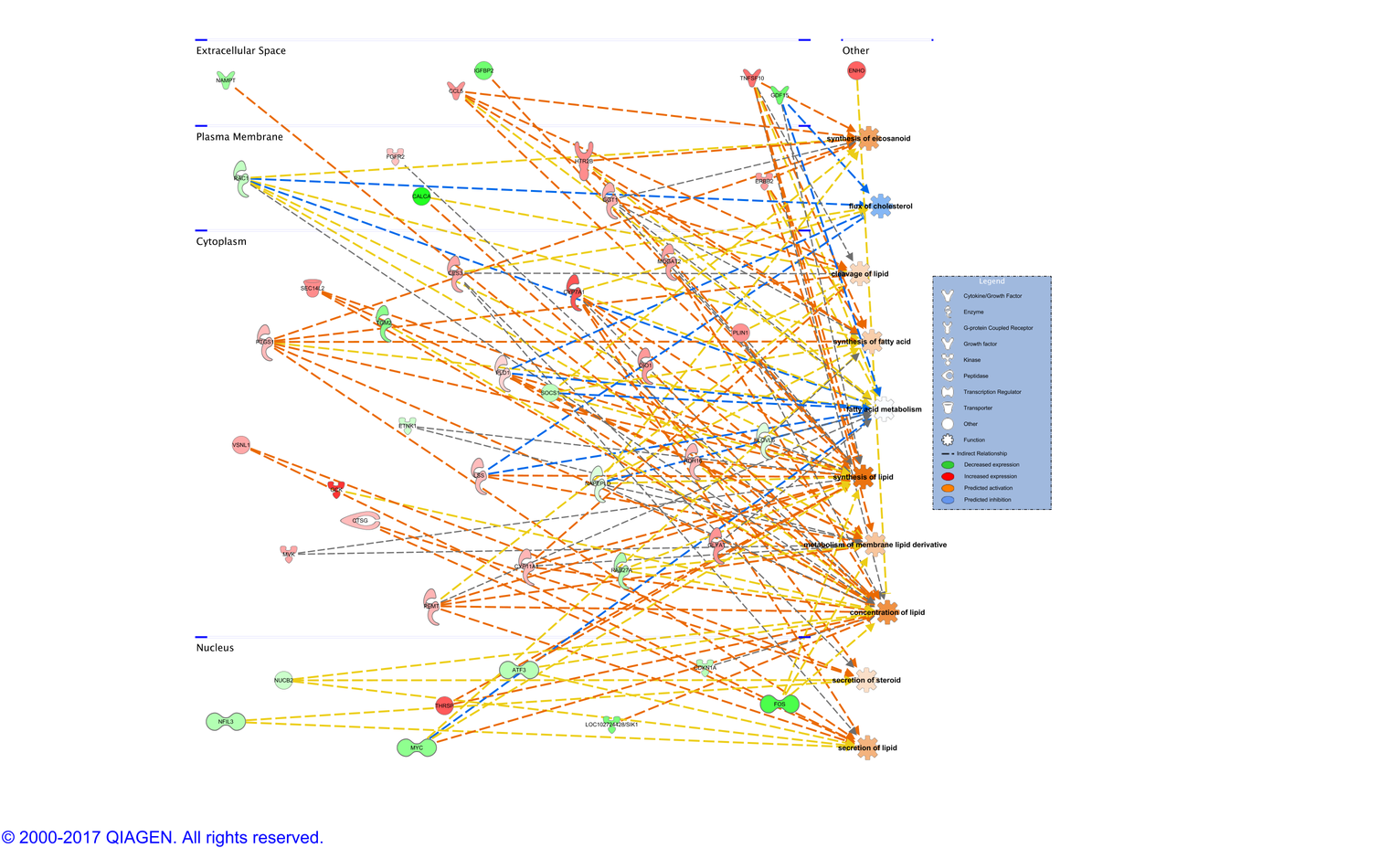

Supplement: S3 Fig — (TIF) [file pone.0189223.s003.tif]

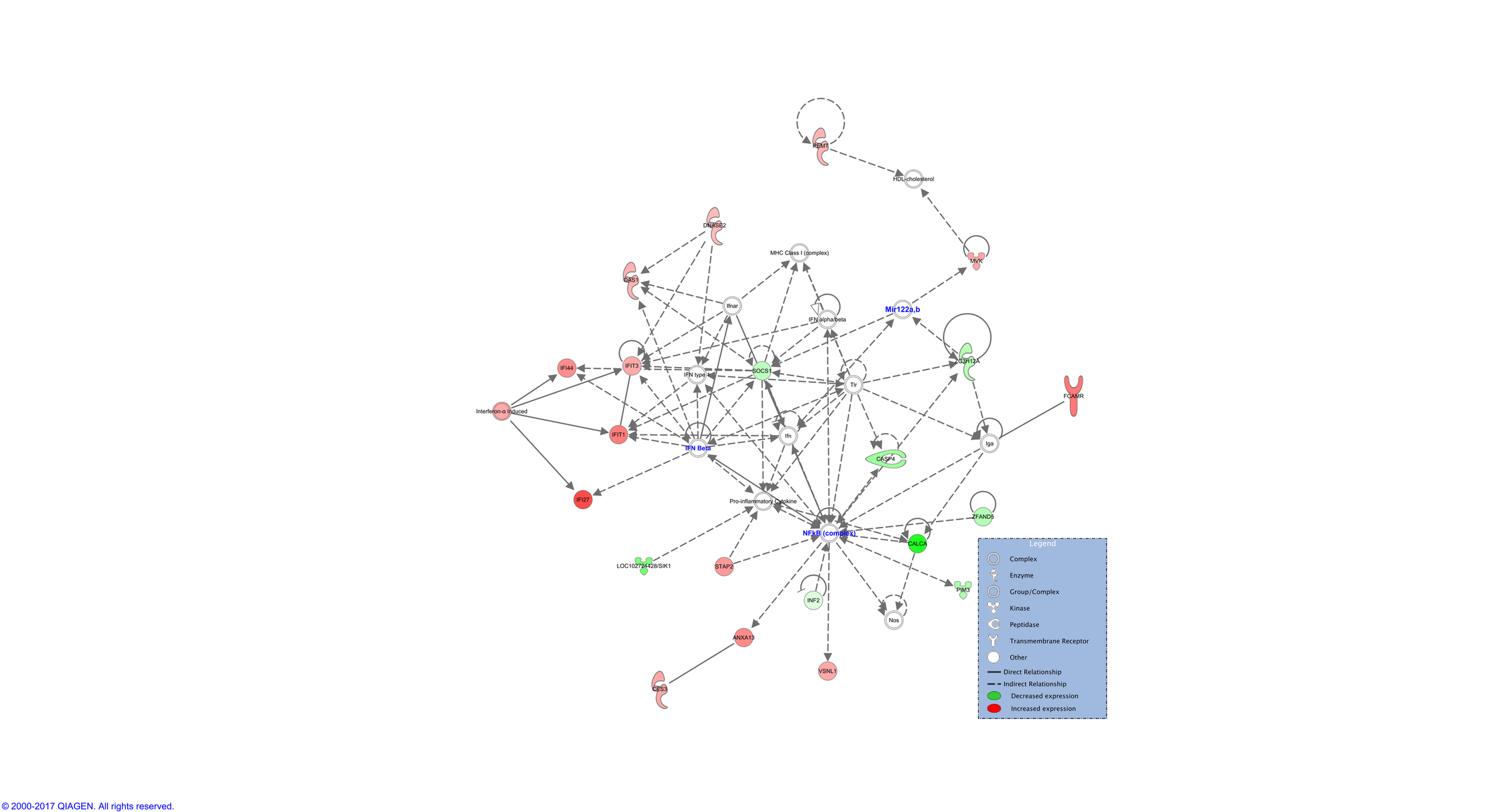

Supplement: S4 Fig — Molecules in bold represent the central nodes. (TIF) [file pone.0189223.s004.tif]

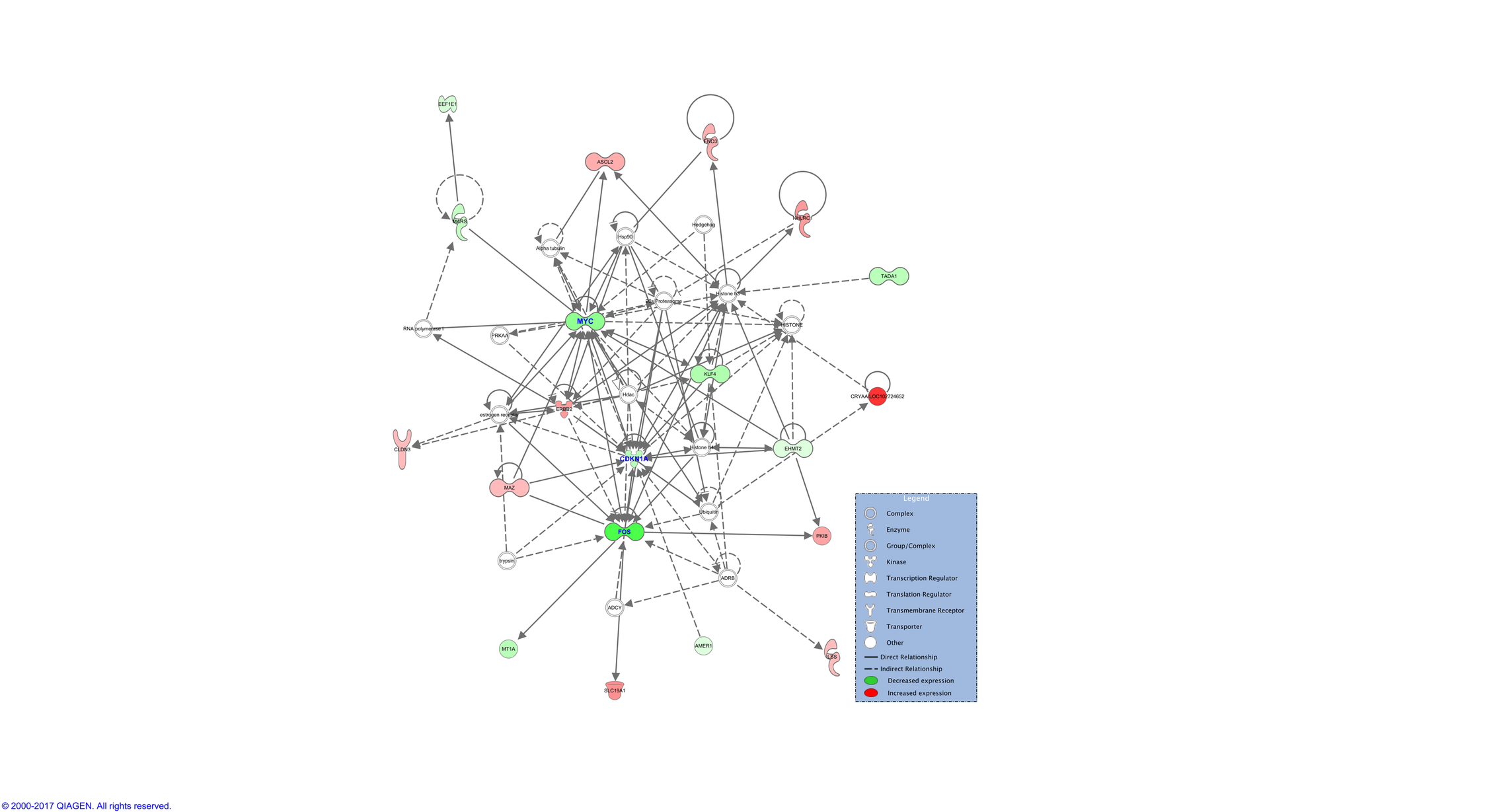

Supplement: S5 Fig — Molecules in bold represent the central nodes. (TIF) [file pone.0189223.s005.tif]

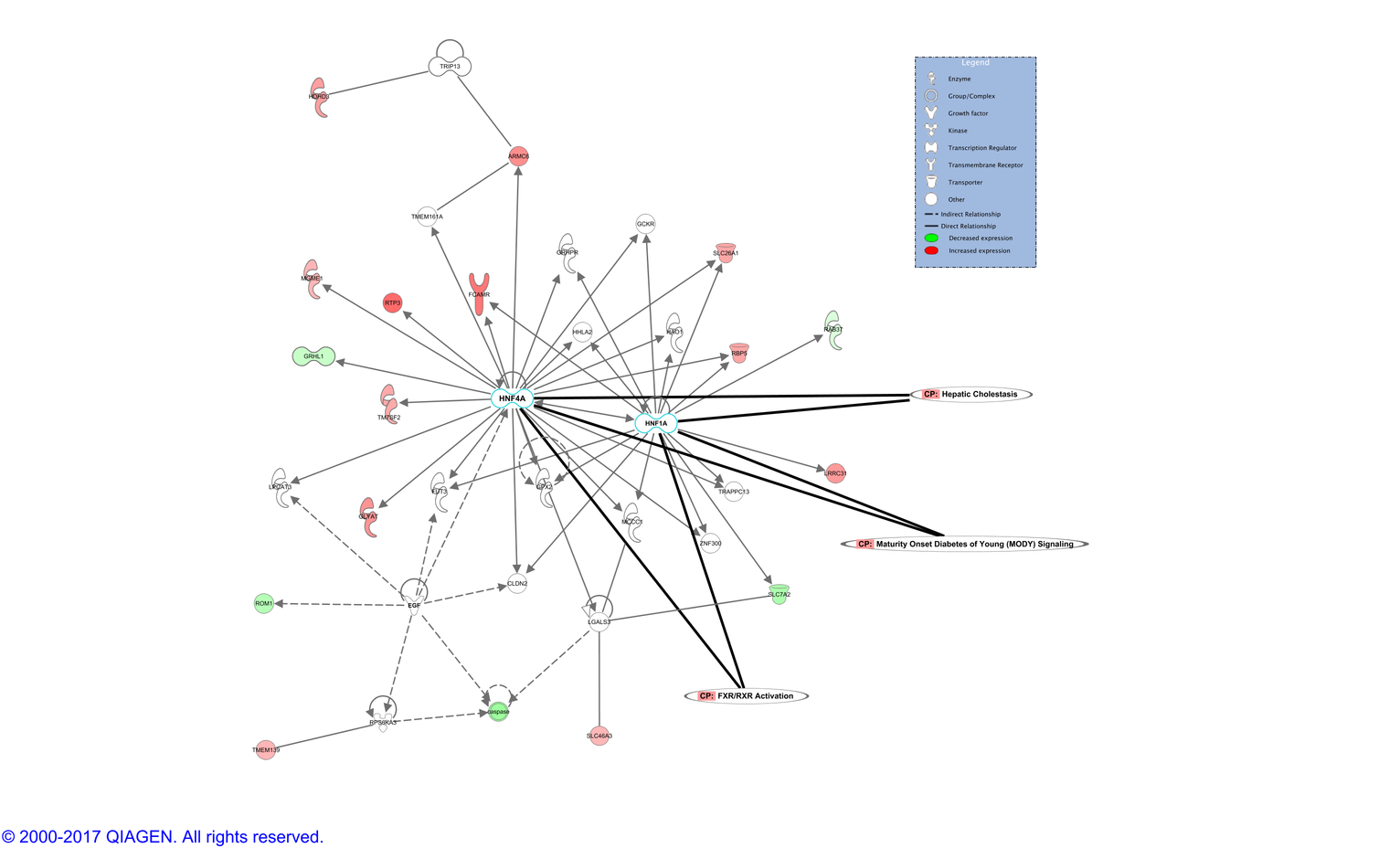

Supplement: S6 Fig — Molecules in bold represent the central nodes. CP = canonical pathway. (TIF) [file pone.0189223.s006.tif]

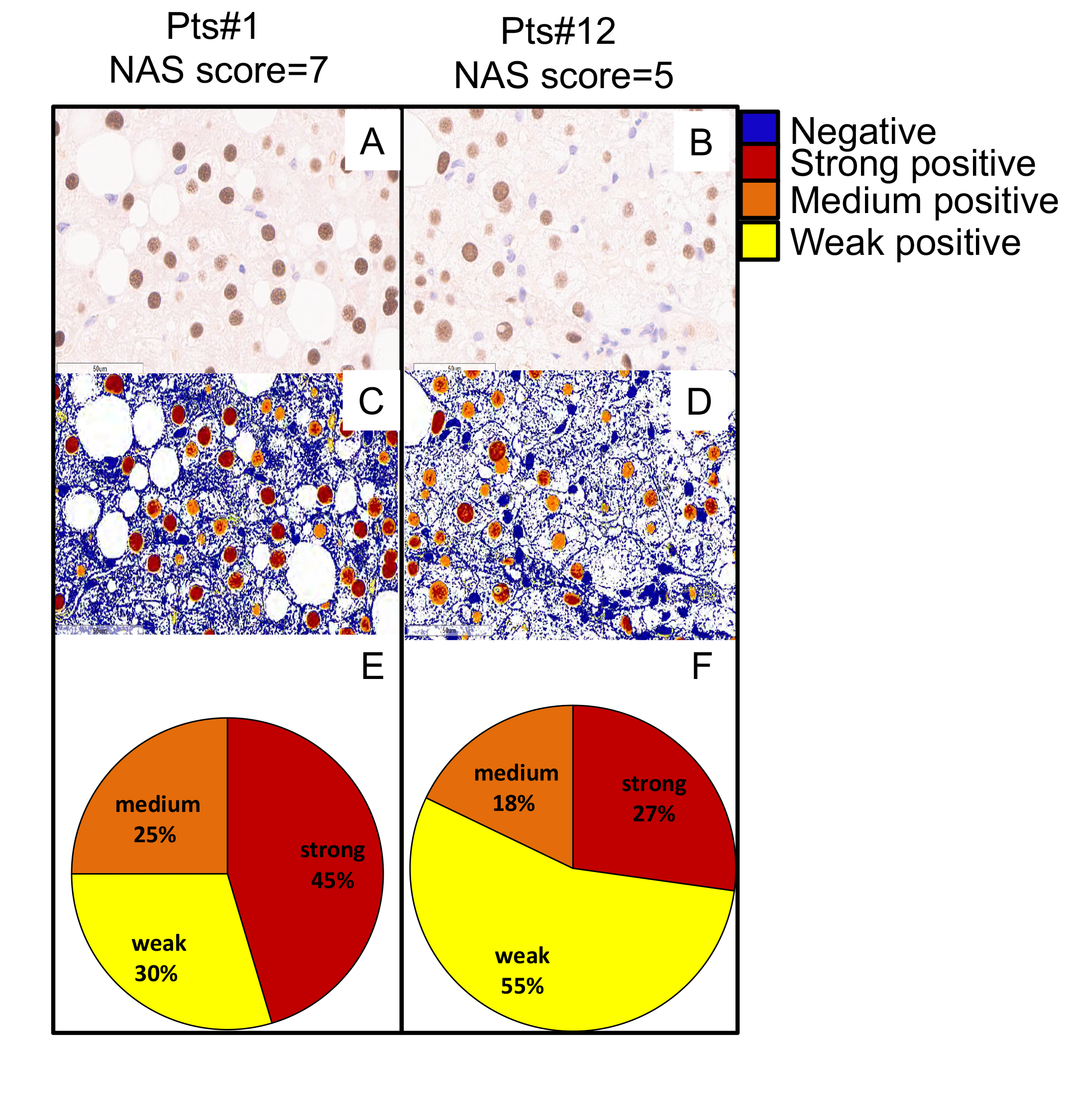

Supplement: S7 Fig — A, B immunohistochemical analysis of liver sections from NASH patients (A, pts#1 NAS score = 7, B pts#12 NAS score = 5), magnification 40X. Sections were stained with HNF4A-DAB and counterstained with hematoxylin. C, D digital markup of sections depicted in column A and B, (C, pts#1 NAS score = 7, D pts#12 NAS score = 5) elaborated by Positive Pixel Count v9 algorithm using Aperio ImageScope image analysis software; blue = negative; yellow = weak positive; orange = positive; brown = strong positive. E, F pie charts represent the digital quantification of percentage of strong, medium and weak positive pixel quantified over the total of positive HNF4A pixel in the whole section (E, pts#1 NAS score = 7; F, pts#12 NAS score = 5). (TIF) [file pone.0189223.s007.tif]
