# Supplementary material for: Systematic integrative analysis of gene expression identifies HNF4A as the central gene in pathogenesis of non-alcoholic steatohepatitis
Source: PLoS One. 2017 Dec 7;12(12):e0189223. doi: 10.1371/journal.pone.0189223 (PMC5720788; doi:10.1371/journal.pone.0189223)
Supplement: S1 Table — (DOCX) [file pone.0189223.s008.docx]

**S1 Table.** Upstream regulators predicted to be inactivated.

| **Upstream Regulator** | **Molecule Type** | **Bias-corrected**  **z-score** | **p-value of overlap** |
| --- | --- | --- | --- |
| hydrogen peroxide | chemical - endogenous mammalian | -3.654 | 6.24E-05 |
| leukotriene D4 | chemical - endogenous mammalian | -2.794 | 1.38E-04 |
| norepinephrine | chemical - endogenous mammalian | -2.472 | 3.72E-03 |
| dihydrotestosterone | chemical - endogenous mammalian | -2.487 | 2.93E-05 |
| Ca2+ | chemical - endogenous mammalian | -2.415 | 2.48E-03 |
| bicuculline | chemical - endogenous non-mammalian | -3.045 | 5.34E-08 |
| tunicamycin | chemical - endogenous non-mammalian | -2.783 | 1.70E-03 |
| trovafloxacin | chemical drug | -3.297 | 1.04E-08 |
| gentamicin | chemical drug | -3.191 | 4.06E-04 |
| lipopolysaccharide | chemical drug | -3.859 | 2.68E-13 |
| fluticasone | chemical drug | -2.9 | 6.86E-08 |
| camptothecin | chemical drug | -2.939 | 3.34E-03 |
| dalfampridine | chemical drug | -3.246 | 1.23E-08 |
| estrogen | chemical drug | -3.09 | 9.48E-06 |
| doxorubicin | chemical drug | -2.896 | 1.09E-05 |
| carboplatin | chemical drug | -2.588 | 1.16E-04 |
| lomustine | chemical drug | -2.725 | 2.84E-04 |
| 5-azacytidine | chemical drug | -2.721 | 9.23E-03 |
| deferoxamine | chemical drug | -2.692 | 1.88E-06 |
| cisplatin | chemical drug | -2.494 | 2.49E-04 |
| phenacetin | chemical drug | -2.739 | 3.72E-05 |
| gentamicin C | chemical drug | -2.721 | 2.64E-04 |
| triamterene | chemical drug | -2.738 | 3.28E-04 |
| allopurinol | chemical drug | -2.568 | 9.95E-04 |
| methylprednisolone | chemical drug | -2.387 | 4.41E-07 |
| paclitaxel | chemical drug | -2.397 | 2.74E-06 |
| vitamin K3 | chemical drug | -2.627 | 5.72E-06 |
| cyclophosphamide | chemical drug | -2.563 | 3.50E-07 |
| CD 437 | chemical drug | -1.707 | 3.89E-02 |
| vitamin D | chemical drug | -2.066 | 1.16E-03 |
| GnRH-A | chemical reagent | -3.608 | 2.02E-12 |
| fenamic acid | chemical reagent | -2.707 | 4.04E-04 |
| 2-bromoethylamine | chemical reagent | -2.717 | 7.93E-05 |
| dithiothreitol | chemical reagent | -2.293 | 9.67E-05 |
| thapsigargin | chemical toxicant | -3.301 | 2.92E-08 |
| hexachlorobenzene | chemical toxicant | -2.734 | 1.64E-04 |
| methyl methanesulfonate | chemical toxicant | -2.743 | 4.18E-05 |
| carbon tetrachloride | chemical toxicant | -2.675 | 1.98E-05 |
| PDGF BB | complex | -4.801 | 1.25E-11 |
| NFkB (complex) | complex | -3.635 | 1.24E-12 |
| PI3K (complex) | complex | -2.825 | 1.13E-05 |
| TCR | complex | -2.588 | 8.96E-05 |
| VitaminD3-VDR-RXR | complex | -2.338 | 7.87E-04 |
| TNF | cytokine | -4.164 | 2.29E-19 |
| IL1B | cytokine | -3.747 | 3.35E-21 |
| IL6 | cytokine | -3.534 | 1.18E-09 |
| EPO | cytokine | -3.173 | 4.72E-03 |
| CSF3 | cytokine | -2.845 | 4.66E-07 |
| LIF | cytokine | -2.578 | 1.29E-05 |
| Il3 | cytokine | -2.383 | 3.77E-04 |
| OSM | cytokine | -2.408 | 9.18E-03 |
| ACKR2 | g-protein coupled receptor | -1.539 | 3.39E-04 |
| ERK | group | -3.589 | 9.81E-06 |
| Mek | group | -2.864 | 1.13E-05 |
| MAP2K1/2 | group | -2.58 | 3.52E-04 |
| EGF | growth factor | -3.523 | 1.49E-10 |
| FGF2 | growth factor | -3.215 | 4.06E-04 |
| TGFB1 | growth factor | -3.088 | 2.41E-07 |
| GDF2 | growth factor | -2.76 | 1.31E-04 |
| TGFB3 | growth factor | -2.619 | 2.67E-03 |
| HGF | growth factor | -2.76 | 1.10E-05 |
| MAPK1 | kinase | -2.295 | 1.54E-08 |
| NR1I2 | ligand-dependent nuclear receptor | -2.337 | 7.47E-05 |
| MYD88 | other | -3.34 | 2.27E-05 |
| F2 | peptidase | -3.634 | 5.79E-07 |
| F7 | peptidase | -2.7 | 9.49E-06 |
| NUPR1 | transcription regulator | -3.447 | 8.32E-07 |
| FOXO4 | transcription regulator | -2.775 | 3.07E-06 |
| STAT3 | transcription regulator | -2.694 | 2.36E-09 |
| RELA | transcription regulator | -2.835 | 1.79E-08 |
| TP53 | transcription regulator | -2.468 | 6.52E-06 |
| TP63 | transcription regulator | -2.437 | 1.54E-04 |
| SMAD4 | transcription regulator | -2.478 | 6.38E-07 |
| EGR1 | transcription regulator | -2.573 | 2.31E-04 |
| CREB1 | transcription regulator | -2.337 | 5.71E-09 |
| PLAG1 | transcription regulator | -2.349 | 1.54E-03 |
