# Supplementary material for: Systematic integrative analysis of gene expression identifies HNF4A as the central gene in pathogenesis of non-alcoholic steatohepatitis
Source: PLoS One. 2017 Dec 7;12(12):e0189223. doi: 10.1371/journal.pone.0189223 (PMC5720788; doi:10.1371/journal.pone.0189223)
Supplement: S2 Table — (DOCX) [file pone.0189223.s009.docx]

**S2 Table.** IPA predicted networks associated with NASH.

| **ID** | **Top Diseases and Functions** | **Score** | **Focus Molecules** |
| --- | --- | --- | --- |
| 1 | Antimicrobial Response, Inflammatory Response, Immunological Disease | 37 | 21 |
| 2 | Cancer, Organismal Injury and Abnormalities, Cellular Development | 35 | 20 |
| 3 | Cellular Movement, Cancer, Cell Death and Survival | 30 | 18 |
| 4 | Cell Morphology, Hematological System Development and Function, Immunological Disease | 26 | 16 |
| 5 | Auditory Disease, Auditory and Vestibular System Development and Function, Organ Morphology | 26 | 16 |
| 6 | Glomerular Injury, Metabolic Disease, Organismal Injury and Abnormalities | 26 | 16 |
| 7 | Cell Morphology, Embryonic Development, Hair and Skin Development and Function | 22 | 14 |
| 8 | Cell-To-Cell Signaling and Interaction, Drug Metabolism, Molecular Transport | 20 | 13 |
| 9 | Immunological Disease, Neurological Disease, Organismal Injury and Abnormalities | 20 | 13 |
| 10 | Cell Death and Survival, Cell Cycle, Cell-To-Cell Signaling and Interaction | 16 | 11 |
| 11 | Nucleic Acid Metabolism, Small Molecule Biochemistry, Cellular Development | 14 | 10 |
| 12 | Amino Acid Metabolism, Molecular Transport, Small Molecule Biochemistry | 12 | 9 |
| 13 | Cellular Assembly and Organization, Hair and Skin Development and Function, Carbohydrate Metabolism | 10 | 8 |
| 14 | RNA Post-Transcriptional Modification, Cellular Assembly and Organization, Cellular Function and Maintenance | 7 | 6 |
| 15 | Cancer, Endocrine System Disorders, Gastrointestinal Disease | 2 | 1 |
