# Supplementary material for: Systematic integrative analysis of gene expression identifies HNF4A as the central gene in pathogenesis of non-alcoholic steatohepatitis
Source: PLoS One. 2017 Dec 7;12(12):e0189223. doi: 10.1371/journal.pone.0189223 (PMC5720788; doi:10.1371/journal.pone.0189223)
Supplement: S3 Table — (DOCX) [file pone.0189223.s010.docx]

**S3 Table.** Distribution of healthy control and NASH samples from GEO data sets.

| **GEO Accession** | **NASH samples** | **Controls** | **Total samples** |
| --- | --- | --- | --- |
| GSE17470 | 7 | 4 | 11 |
| GSE24807 | 12 | 5 | 17 |
| GSE37031 | 8 | 7 | 15 |
| GSE89632 | 19 | 24 | 43 |
