# Supplementary material for: Systematic integrative analysis of gene expression identifies HNF4A as the central gene in pathogenesis of non-alcoholic steatohepatitis
Source: PLoS One. 2017 Dec 7;12(12):e0189223. doi: 10.1371/journal.pone.0189223 (PMC5720788; doi:10.1371/journal.pone.0189223)
Supplement: S4 Table — (DOCX) [file pone.0189223.s011.docx]

**S4 Table.** List of DEGs and the mean log_2_(FC). FC = fold change.

| **Gene symbol** | **Mean(log_2_FC)** | **Gene symbol** | **Mean(log_2_FC)** | **Gene symbol** | **Mean(log_2_FC)** |
| --- | --- | --- | --- | --- | --- |
| CALCA | -5.34492 | ROM1 | -1.74378 | UPF3B | -0.0171797 |
| RASD1 | -4.39417 | RPRD1B | -1.73277 | GOLM1 | 0.268293 |
| FOS | -4.30618 | EBLN2 | -1.72101 | PDE11A | 0.710853 |
| GDF15 | -3.46281 | ZC3H12A | -1.72014 | PLD1 | 0.771377 |
| IGFBP2 | -3.40442 | CDKN1A | -1.71967 | PDE6D | 0.852374 |
| SIK1 | -3.16033 | ATF3 | -1.704 | TMEM19 | 0.902073 |
| IGFBP1 | -3.13986 | MT1A | -1.69697 | SUOX | 0.906671 |
| DHRS13 | -3.13268 | TADA1 | -1.63029 | NUDT6 | 0.933704 |
| IER3 | -2.93569 | RHOB | -1.62741 | FAM69B | 0.976631 |
| CSRNP1 | -2.90626 | SOCS1 | -1.57836 | PRAP1 | 0.984175 |
| TGM2 | -2.77578 | THBS1 | -1.57094 | GFRA1 | 1.00167 |
| CYR61 | -2.73317 | ELF3 | -1.51442 | MOGAT3 | 1.00629 |
| MYC | -2.66665 | BCL3 | -1.49566 | ART4 | 1.02881 |
| KLF6 | -2.36244 | PRRX1 | -1.47061 | ALDH3B1 | 1.05931 |
| SIPA1L2 | -2.35582 | RAC1 | -1.45283 | FGFR2 | 1.07624 |
| NAMPT | -2.34157 | ETS2 | -1.45148 | PYROXD2 | 1.09147 |
| GADD45B | -2.33624 | GNL3 | -1.39979 | ZNF616 | 1.0931 |
| CCL20 | -2.31893 | MARS | -1.37616 | GJB1 | 1.12549 |
| CASP4 | -2.3017 | GRHL1 | -1.30446 | LYNX1 | 1.14807 |
| ZFP36 | -2.2449 | BTG2 | -1.29439 | RGS16 | 1.1553 |
| SPSB1 | -2.17644 | NUCB2 | -1.22672 | LSS | 1.17295 |
| UPP1 | -2.1593 | ETNK1 | -1.18995 | CTSG | 1.17877 |
| IVNS1ABP | -2.05943 | HTRA1 | -1.13189 | MAZ | 1.18037 |
| SOCS2 | -2.02659 | EEF1E1 | -1.01648 | DNASE2 | 1.18853 |
| PHLDA1 | -1.99744 | RAB37 | -0.858622 | CLDN3 | 1.19247 |
| PIM3 | -1.99221 | INF2 | -0.793338 | B4GAT1 | 1.19438 |
| SLC7A2 | -1.9535 | DET1 | -0.704545 | PTGS1 | 1.19658 |
| KLF4 | -1.90305 | NAPEPLD | -0.681659 | FBXO8 | 1.20893 |
| MTUS1 | -1.87856 | STAG3L5P | -0.673413 | HFE | 1.21023 |
| NCR3LG1 | -1.87604 | AMER1 | -0.643583 | FBLN7 | 1.22838 |
| NFIL3 | -1.83453 | DUSP10 | -0.616352 | GZMH | 1.24809 |
| RAB27A | -1.83236 | FZR1 | -0.609891 | SLC46A3 | 1.25515 |
| ZFAND5 | -1.821 | C19orf12 | -0.505553 | TMEM139 | 1.26852 |
| HAL | -1.80571 | EHMT2 | -0.440971 | CYP11A1 | 1.27256 |
| IER2 | -1.74631 | ELOVL6 | -0.361088 | RDH16 | 1.2783 |
| MGME1 | 1.28427 | MOGAT2 | 1.62487 | ANXA13 | 2.04758 |
| CX3CR1 | 1.30031 | TMEM98 | 1.63099 | C2orf82 | 2.06173 |
| SLC25A20 | 1.30187 | ORAI3 | 1.65745 | CD160 | 2.06914 |
| PEMT | 1.30981 | HDHD3 | 1.6635 | SGCB | 2.08797 |
| BTN3A2 | 1.31783 | SMO | 1.67876 | LTB | 2.09601 |
| OAS1 | 1.34344 | MPLKIP | 1.68029 | TCEA2 | 2.13246 |
| GINS2 | 1.34508 | SLC22A7 | 1.68892 | NOL4 | 2.13294 |
| RNLS | 1.34662 | GRAMD1C | 1.70956 | IFIT1 | 2.29452 |
| ENO3 | 1.39818 | DAO | 1.72876 | APOL3 | 2.35492 |
| ATP6V0E2 | 1.39933 | DSG1 | 1.76633 | ZGPAT | 2.37468 |
| UNC93A | 1.41366 | LRRC31 | 1.76944 | FCAMR | 2.37819 |
| AJUBA | 1.41384 | NHLRC1 | 1.77114 | GPD1 | 2.49116 |
| GGT1 | 1.42017 | ERBB2 | 1.7739 | TNFSF10 | 2.49441 |
| ASCL2 | 1.43636 | STAP2 | 1.78169 | ACP5 | 2.5088 |
| ZNF385B | 1.44502 | DIO1 | 1.78803 | CES5A | 2.54608 |
| TM7SF2 | 1.49241 | COQ10A | 1.82262 | RTP3 | 2.59234 |
| INHBE | 1.49638 | BTN3A3 | 1.84558 | QPRT | 2.62045 |
| IFIT3 | 1.50195 | GLYAT | 1.86713 | ENHO | 2.76107 |
| ANKRD29 | 1.51752 | CCL5 | 1.86756 | RAB26 | 2.84426 |
| VSNL1 | 1.51846 | PLIN1 | 1.89667 | THRSP | 2.89792 |
| CES3 | 1.53153 | SLC19A1 | 1.94879 | DGCR5 | 2.98815 |
| SLC26A1 | 1.53559 | GUCY1A3 | 1.95031 | CYP7A1 | 2.98874 |
| LGI1 | 1.5482 | ARMC6 | 1.95914 | IFI27 | 3.16145 |
| MVK | 1.55096 | CNTNAP2 | 1.98959 | SEC14L3 | 3.38333 |
| RBP5 | 1.55895 | ANKRD55 | 1.99101 | FNDC5 | 3.43172 |
| CASC10 | 1.55926 | MEP1B | 1.99838 | CRYAA | 3.55326 |
| PKIB | 1.59079 | HTR2B | 2.00276 | GCK | 3.5845 |
| CCL15 | 1.60659 | IFI44 | 2.00284 |  |  |
| MATK | 1.61748 | SEC14L2 | 2.01419 |  |  |
