# Supplementary material for: Systematic integrative analysis of gene expression identifies HNF4A as the central gene in pathogenesis of non-alcoholic steatohepatitis
Source: PLoS One. 2017 Dec 7;12(12):e0189223. doi: 10.1371/journal.pone.0189223 (PMC5720788; doi:10.1371/journal.pone.0189223)
Supplement: S5 Table — (DOCX) [file pone.0189223.s012.docx]

**S5 Table.** Clinical information of NASH and Control patients from GEO studies.

| **PMID** | **Steatosis**  **(% hepatocytes)** | **Gender**  **NASH** | **Controls** | **BMI** | **Steatosis grade (%)** | **IR** |
| --- | --- | --- | --- | --- | --- | --- |
| 23492103 | N/A | N/A | N/A | N/A | N/A | N/A |
| 21931690 | NA | F=3; M=9 | F=1; M=4 | average 32  (22-45) | N/A | 4.2(1.2-8.4) HOMA |
| 20221393 | N/A | F=2; M=5 | F=1; M=3 | 35.0±2.9 (NASH); 19.1±1.6(control) | N/A | 6.0±0.5 |
| 25581263 | 45 | F=10; M=9 | F=13; M=11 | 31.9 (NASH); 26.1(control) | grade 1=31.6; grade 2=36.8 ; grade 3=31.6 | N/A |
